# A large gastrointestinal stromal tumor of the duodenum treated by partial duodenectomy with Roux-en-Y duodenojejunostomy: a case report

**DOI:** 10.1186/1752-1947-7-184

**Published:** 2013-07-15

**Authors:** Ouadii Mouaqit, Leila Chbani, Khalid Maazaz, Afaf Amarti, Khalid Ait Taleb, Abdelmalek Oussaden

**Affiliations:** 1 Surgery Department, University Hospital Hassan II, Fez, Morocco; 2 Department of Pathology, University Hospital Hassan II, Fez, Morocco

**Keywords:** Duodenojejunostomy, Duodenum, Gastrointestinal stromal tumors, Surgery

## Abstract

**Introduction:**

Duodenal gastrointestinal stromal tumors are uncommon and a relatively small subset of gastrointestinal stromal tumors whose optimal surgical procedure has not been well defined. Because submucosal spread and local lymph node involvement are infrequent in gastrointestinal stromal tumors, wide margins with routine lymph node dissection may not be required. Various surgical procedures for duodenal gastrointestinal stromal tumor, pancreatoduodenectomy, pancreas-sparing duodenectomy, segmental duodenectomy, or local resection, have been described depending on the size and exact site of the lesion.

**Case presentation:**

We present the case of a 65-year-old African woman with a giant gastrointestinal stromal tumor involving the second and third portion of the duodenum successfully treated by partial duodenectomy with duodenojejunostomy. This surgical technique is ideal when a gastrointestinal stromal tumor does not involve the ampulla because it avoids a pancreatoduodenectomy, and has not been previously described for the management of this malignancy. Duodenal gastrointestinal stromal tumor should be suspected in any patient with a duodenal wall mass.

**Conclusions:**

Gastrointestinal stromal tumors of the duodenum should be suspected in any patient with a duodenal wall mass. Extramural growth and central ulceration with or without bleeding should alert the endoscopist to the possibility of a duodenal gastrointestinal stromal tumor diagnosis.

## Introduction

Only 3% to 5% of gastrointestinal stromal tumors (GISTs) are located in the duodenum. They are associated with an increased risk of fatal gastrointestinal bleeding, which is a primary manifestation [1,2]. A small intestinal GIST can occur anywhere along the length of the bowel and can be multiple. The duodenum is involved in about 10% to 20% of small intestinal GISTs [3]. Although duodenal GISTs are similar pathologically to those involving other organs, they do have some peculiar features. GISTs in the duodenum pose particular challenges for diagnosis and management. We describe the case of a large duodenal GIST including its presentation, diagnosis, and the type of surgery performed, as well as a review of issues related to GISTs in the duodenum.

## Case presentation

**Figure 1:**
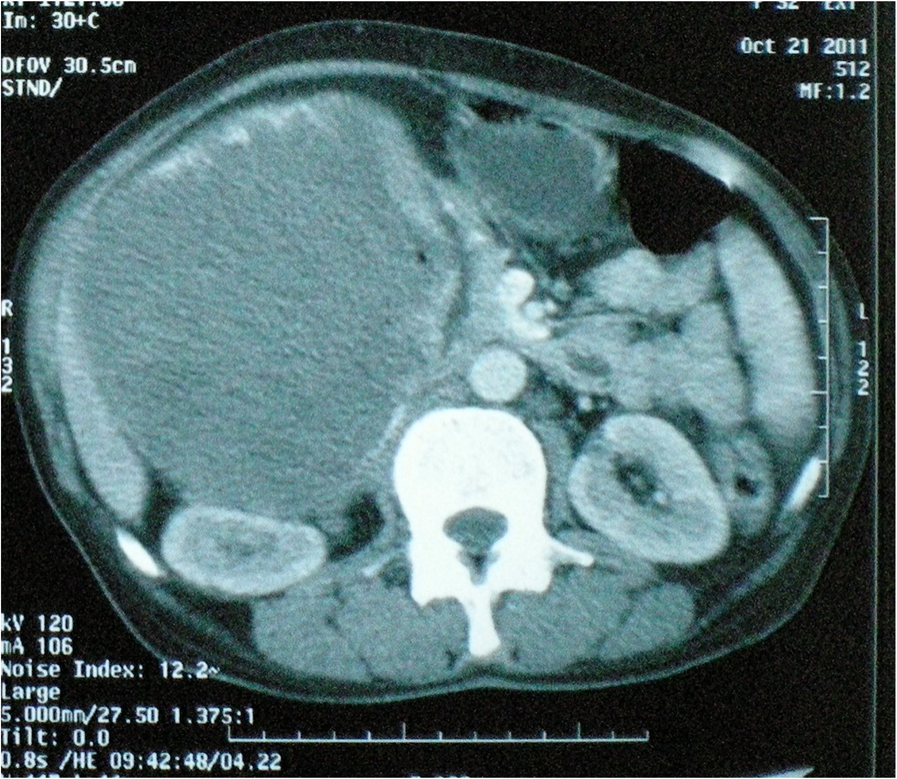
Abdominal computed tomography showing the large retropancreatic tumor.

**Figure 2:**
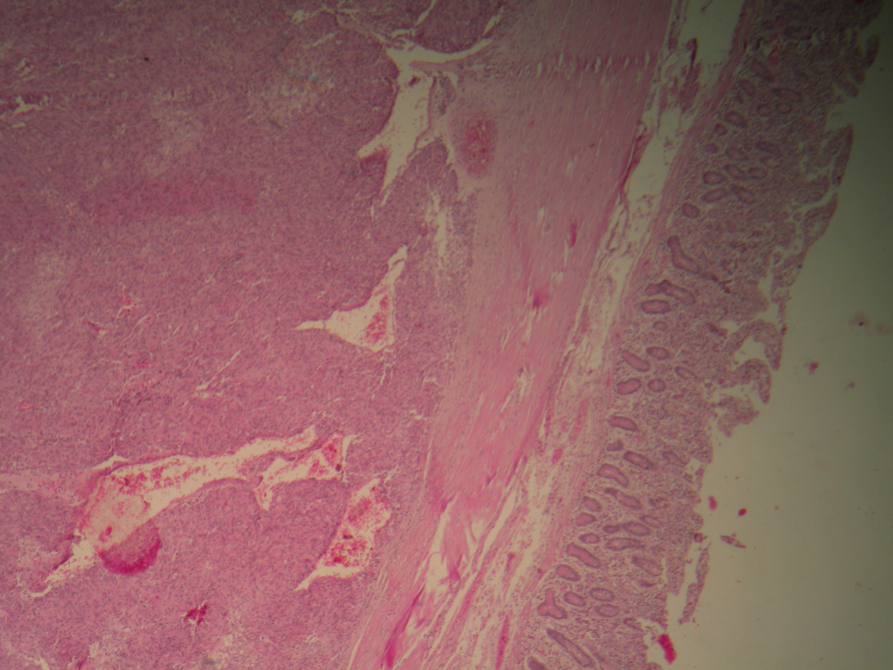
**Histological findings of the tumor.** Histopathology showing spindle-shaped tumor arranged in palisading pattern.

**Figure 3:**
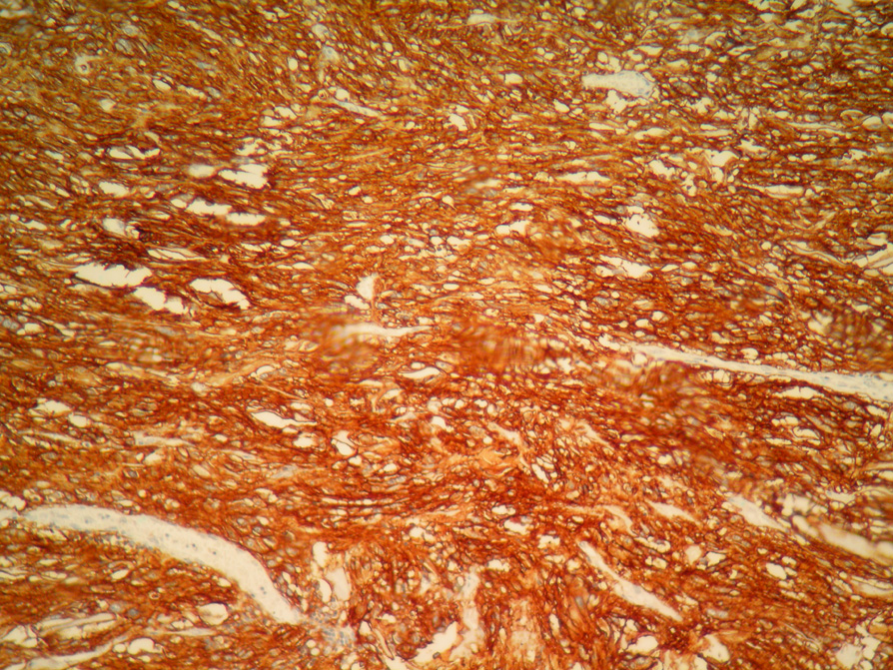
Immunohistochemistry showing positivity for CD117.

**Figure 4:**
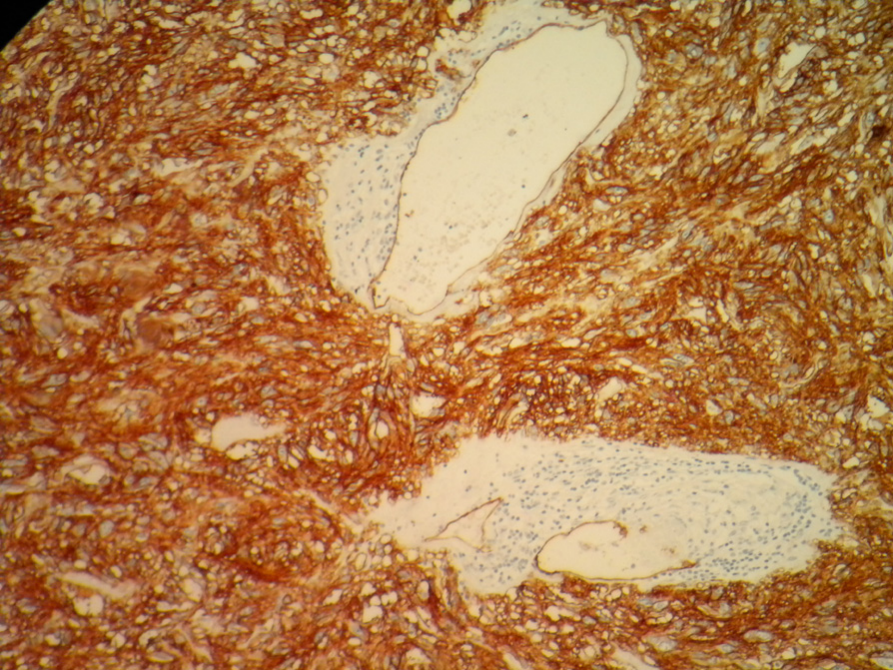
**Immunohistochemistry.** The tumor cells react positively for CD34.

**Figure 5:**
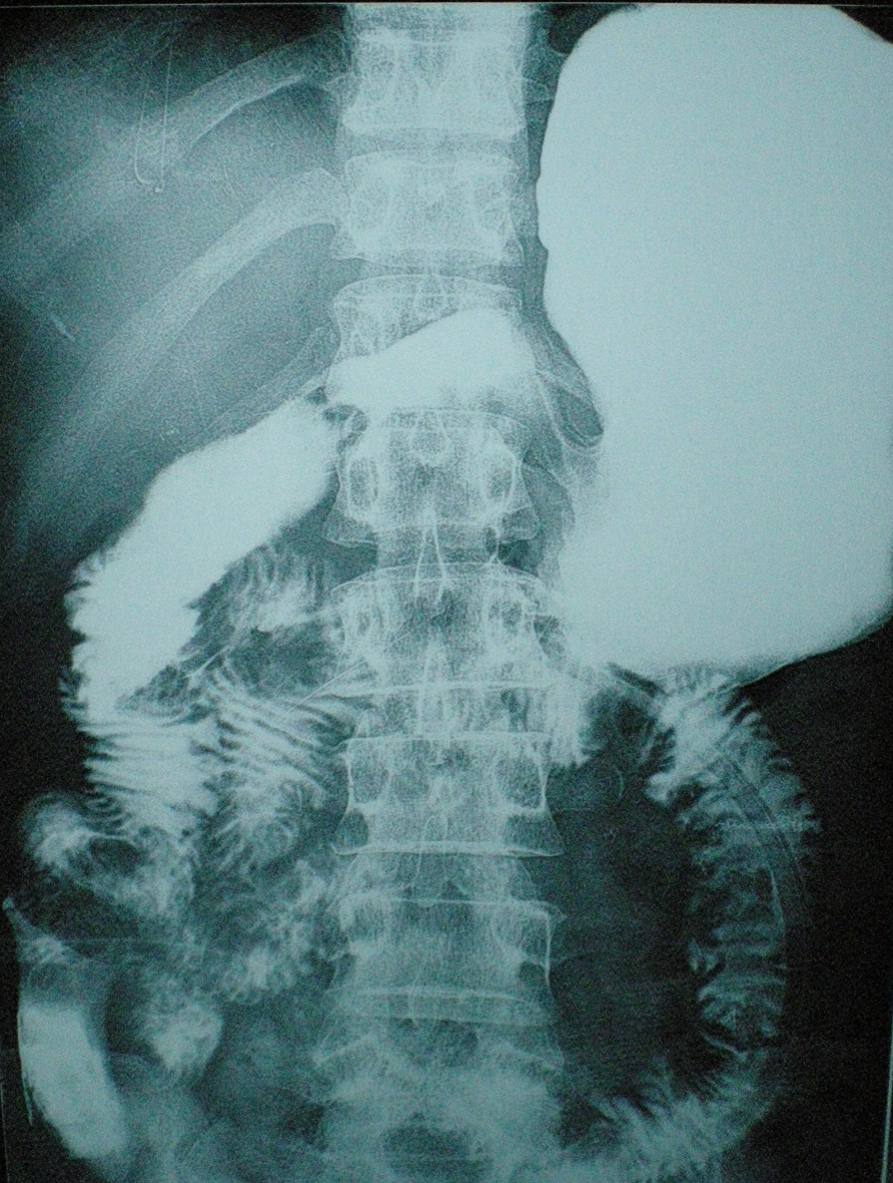
The postoperative digestive opacification.

A 65-year-old African woman presenting with abdominal pain was referred to our hospital. Her medical history and family history were unremarkable. She had no history of previous abdominal surgery. On examination she looked healthy with no clinical jaundice or pallor. An abdominal examination revealed a large upper abdominal mass with thinned overlying skin. It had minimal mobility and was not tender. The rest of the examination was normal. Her hemoglobin, on admission, was 7.0g/dL. She was transfused and underwent an esophagogastroduodenoscopy, which revealed a submucosal tumor at D2 and D3. A biopsy was obtained but was reported as nonspecific. A computed tomography (CT) scan of the abdomen revealed a 12×13cm retroperitoneal mass in the region of the head of the pancreas (Figure 1). There was no evidence of metastases to her liver or lung. From these radiographic findings, we diagnosed a submucosal tumor of the duodenum. The patient underwent an elective exploratory laparotomy. No evidence of local invasion of the pancreas or of distant metastases was found. The tumor had greatly decreased in size, and it was thought that the liquid of the tumor had probably emptied into the duodenum through a fistula between the tumor and the duodenum. Considering that the pancreas and major papilla were not involved, a local resection was performed, with a 1cm disease-free margin. A retrocolic Roux-en-Y loop was then created and the edges of the defect in the duodenum joined to the jejunal limb of the Roux-loop by a hand-stitched side-to-side duodenojejunostomy anastomosis using a 3-0 Vicryl. In addition, a resection of the right hemicolon was performed due to tumor infiltration of the right curvature of the colon. An ileotransversostomy was performed to reconstruct the gastrointestinal passage. The operative time was 200 minutes and estimated blood loss was 100mL. Microscopic examination with hematoxylin and eosin staining of the tumor showed spindle shaped and epithelioid cells with mild nuclear pleomorphism (Figure 2). Immunohistochemistry revealed that the cells strongly expressed CD117 (Figure 3), with focal expression of CD34 (Figure 4). Therefore, the final histology was consistent with the diagnosis of a duodenal GIST. Based on the above findings, the tumor was finally diagnosed as a GIST with high-grade malignancy originating from the duodenum. A molecular genetic analysis for KIT protein mutation was not performed because of its unavailability at our institute. After the operation, the postoperative digestive opacification showed no digestive fistula (Figure 5). The patient was treated with imatinib. She was doing very well with no evidence of disease recurrence when she was last seen 24 months after her operation.

## Discussion

GISTs are defined as mesenchymal tumors arising from the gastrointestinal wall, mesentery, omentum or retroperitoneum. GISTs can be located anywhere in the gastrointestinal tract. The most common sites are stomach (40% to 60%) and small intestine (30% to 40%) [4]. The mean age of patients with GIST is 53 years. Only about 5% of GIST patients are younger than 30 years [4]. GISTs of the duodenum make up only 4.5% of all GISTs [5] and therefore represent a rare tumor entity. Duodenal GISTs are mainly located in the second portion of the duodenum (42 out of 156) and about half of them are malignant [4]. The woman described in this case report presented with a duodenal GIST as another rare GIST manifestation. On presentation, 41% to 47% of malignant GISTs are metastatic [6]. These tumors grow expansively without being invasive and sometimes metastasize to the liver or recur locally. A duodenal GIST is usually asymptomatic when small in size and can reach a large size before causing any symptoms. As the tumor enlarges it causes variable symptomatology. The most common presentation is gastrointestinal bleeding which may be chronic and mild or sudden and massive [6]. Although our patient had a large tumor, she had mild anemia. The next most common presentations are abdominal discomfort, pain and swelling [7]. Compared to other tumor localizations duodenal GISTs manifest with tumor-associated bleeding in 75% compared to 54% stomach and 28% ileojejunal. In contrast to other localizations duodenal GISTs are thus associated with a dramatically increased risk of upper intestinal bleeding [8]. Diagnosis can be made with upper gastrointestinal endoscopy [6]. The tumor is usually exophytic, and appears as a submucosal swelling. Sometimes it presents only as an ulcer, as in our case. The biopsy should be deep, but may not always be diagnostic. Endoscopic ultrasound can help in delineating the submucosal tumor. Alternative diagnostic means include CT, magnetic resonance imaging (MRI), barium study or ultrasonography [9]. However, CT and MRI seem to be the best imaging modalities for assessment of the primary lesion and detection of metastases [10]. However, CT scans are not always helpful in specifying the origin of the mass. In several cases reported in the literature, the mass was misdiagnosed as arising from the head of the pancreas [11]. There is currently uniform agreement that the surgical treatment of choice for GISTs is resection of the tumor with clear surgical margins including adjacent organs as necessary [10]. As local and regional lymph node involvement is infrequent in GIST, routine lymph node dissection is not advocated [2,3,7,9] and limited resection is frequently performed. The surgical choice depends not only on the size of the tumor but also on the location in the duodenal wall and the relation to the ampulla of Vater [10,12,13]. Patients with duodenal GISTs close to papilla of Vater should be treated by pancreatoduodenectomy. Various techniques of limited resection for duodenal GISTs have been advocated depending on the site and the size of the tumors. Wedge resection with primary closure can be performed for small lesions if the resulting lumen is adequate and the ampulla can be preserved [14,15]. Segmental duodenectomy with side-to-end or end-to-end duodenojejunostomy can be performed for larger tumors located at the third and fourth portion of the duodenum [14]. Partial duodenectomy with Roux-en-Y duodenojejunostomy can be performed for larger tumors involving the antimesenteric border of the second and third portion of the duodenum [16]. Although a limited operation procedure, such as wedge or segmental resection, is relatively simple to perform, there is a risk of subsequent anastomotic leakage or stenosis development, as well as later tumor recurrence in patients treated by limited operation. By contrast, pancreatoduodenectomy as a treatment for duodenal GISTs can provide a wider surgical margin but may be associated with excessive morbidity, especially in patients with a tumor of low-grade malignancy [17]. It is not clear what the optimal surgical margin should be, but a negative one is essential to prevent local recurrence of the tumor. No lymph node dissection is required because they are very unlikely to be involved [14,18]. The outcome depends on the pathological features of the tumor and the completeness of surgical resection. Large tumors with high mitotic counts behave much worse than small tumors with low mitotic counts, which are considered benign [11]. Local recurrence is higher in tumors not completely removed or with a positive microscopic margin. After a review of recent literature the duodenal tumor localization in our case is thus associated with a better prognosis, but with an increased bleeding probability [8]. These results are in line with the authors' opinion that primary surgery could be the safest therapeutic option for a GIST of this localization. Beside the increased risk of tumor bleeding, caused by the localization, neoadjuvant imatinib therapy would additionally lead to a higher percentage of patients with tumor bleeding. For our patient, there was no indication of adjuvant treatment because the patient presented with severe anemia due to tumor bleeding.

## Conclusions

Duodenal GIST should be suspected in any patient with a duodenal wall mass. Extramural growth and central ulceration with or without bleeding should alert the endoscopist to the possibility of this diagnosis. There is more than one surgical approach available, but the absolute requirement is complete surgical excision. Preoperative imatinib mesylate can be considered in unresectable or borderline resectable cases.

### Consent

Written informed consent was obtained from the patient for publication of this manuscript and accompanying images. A copy of the written consent is available for review by the Editor-in-Chief of this journal.

## Abbreviations

CT: Computed tomography; GIST: Gastrointestinal stromal tumor; MRI: Magnetic resonance imaging.

## Competing interests

The authors declare that they have no competing interests.

## Authors’ contributions

All of the authors were involved in the preparation of this manuscript. OM performed the operation and revised the manuscript. LC described histological finding and was involved in drafting the manuscript. All authors read and approved the final manuscript.
